# Supplementary material for: Farmers Views on the Implementation of On-Farm Emergency Slaughter for the Management of Acutely Injured Cattle in Ireland
Source: Animals (Basel). 2023 Jan 28;13(3):450. doi: 10.3390/ani13030450 (PMC9913314; doi:10.3390/ani13030450)
Supplement: Supplementary file 1 [file animals-13-00450-s001.zip › animals-2111837-supplementary.pdf]

Survey on the Managing of Acutely Injured Cattle /Farmers

Start of Block: Consent

Q1 ***A questionnaire-based survey on the management of acutely injured cattle in the Republic of Ireland.*** *Dear Sir/Madam, I am a Veterinarian working in Veterinary Public Health as a Veterinary Officer with Mayo County Council. I am conducting a project with Associate Professor Alison Hanlon, UCD School of Veterinary Medicine, Assistant Professor Flavia Santos, UCD School of Psychology and Dr. Aideen McKevitt, UCD School of Agriculture and Food Science. The survey should be answered by cattle farmers, both beef and dairy. The survey focuses on the management of acutely injured cattle in the Republic of Ireland. An acute injury is an injury that is severe, causes acute pain, has a sudden onset, is usually associated with a traumatic event and is commonly locomotory. The overall aim of the project is to determine how acutely injured cattle are managed in Ireland. The survey considers three areas: 1.* The methods used by farmers' to manage acutely injured cattle. *2. The farmers' opinions and experience in relation to on farm emergency slaughter. 3. The farmers' opinions on the rules and policies in relation to on farm emergency slaughter.*

All data will be anonymised and stored securely in compliance with UCD rules and regulations. The survey will take approximately 8 - 10 minutes and you must consent to participate in the survey. The survey closes on 21st May. Thanking you, Yours sincerely Paul McDermott, MVB, MSc (VPH), MRCVS. paul.mc-dermott.1@ucdconnect.ie

- ○ Yes, I consent
- ○ No, I do not consent

---

*

Q2 What is your age?

____

Q3 Gender

- ☐ Male
- ☐ Female
- ☐ Other

Q4 Education

- ○ Primary
- ○ Secondary
- ○ Third Level

Q5 My main farming activity is

- ○ Beef
- ○ Dairy
- ○ Other (please specify) ____

---

*

Q6 What is your herd size?

____

Q7 In what county do you farm?

____

____

Q8 Have you participated in Knowledge Transfer Programmes?

- ◯ Yes
- ◯ No

Q9 **How many** acutely injured cattle have you had from 1 Jan to 31 Dec 2020.
An acute injury is an injury that is severe, causes acute pain, has a sudden onset and is usually associated with a traumatic event.

Q10 If 0, when did you last have an acutely injured animal (month/year)?

Skip To: Q14 If Condition: If 0, when did you last hav... Is Equal to 0. Skip To: The nature of the injury of the last ....

Q11
Acutely Injured Statistics

*On-farm emergency slaughter (**OFES**) refers to the on-farm slaughter of healthy cattle that has suffered an accident.*
Casualty Slaughter refers to the slaughter of an injured cattle that has been deemed fit for transport to the abattoir under Veterinary Certification.

The number of acutely injured cattle on my farm that **OFES** was performed on in 2020 was (numeric value)

____

Q12 The number of acutely injured cattle on my farm that were transported to an abattoir for **casualty slaughter** in 2020 was (numeric value)

____

*

Q13 The number of acutely injured cattle on my farm that required **euthanasia** in 2020 was (numeric value)

____

Q14 The **nature of the injury** of the last three acutely injured cattle on my farm was

- ☐ 1 ____
- ☐ 2 ____
- ☐ 3 ____

Q15 Who commonly performs **euthanasia** if required on your farm?

- ◯ Your vet
- ◯ Your knackery
- ◯ Other (please specify) ____________________

---

*

Q16 **The number** of abattoirs I am aware of within a 100 Km radius of my farm that provide the service of **OFES** is (numeric value)

____

Q17 My **knowledge about** the management of acutely injured cattle is informed by (tick all that apply)

- ☐ Guidelines
- ☐ Regulations
- ☐ Other farmers
- ☐ My vet
- ☐ Farming organisations
- ☐ Others (specify) ____________________

Q18 **My experience** about the procedure known as **OFES** on a scale of 0-10 is, where **0 is extremely bad** and **10 is extremely good**

0	1	2	3	4	5	6	7	8	9	10

Number ()	

Q19 **My opinion** about the procedure known as **OFES** on a scale of 0-10 is, where **0 is extremely negative** and **10 is extremely positive**

	0	1	2	3	4	5	6	7	8	9	10
Number ()											

Q20 **Three positive aspects of OFES**, in relation to animal welfare, as a method of dealing with acutely injured cattle are

- [ ] 1 ______________________________
- [ ] 2 ______________________________
- [ ] 3 ______________________________

Q21 **Three negative aspects of OFES**, in relation to animal welfare, as a method of dealing with acutely injured cattle are

- [ ] 1 ______________________________
- [ ] 2 ______________________________
- [ ] 3 ______________________________

Q22 I would recommend the following **three changes** to the current **OFES** procedure

☐ 1 ____________________

☐ 2 ____________________

☐ 3 ____________________

Q23 What matters have you **discussed with your vet** about the management of acutely injured cattle

____

____

____

____

____

Q24 What matters have you **discussed with other farmers** about the management of acutely injured cattle

____

____

____

____

____

Q25 Using a scale of 0-10, **the financial impact** on you of using **OFES** to manage acutely injured cattle is,
where **0 represents the lowest impact** and **10 represents the highest financial impact**

0 1 2 3 4 5 6 7 8 9 10

Number ()	

Q26 Using a scale of 0-10, the **financial impact** on you of **euthanising** acutely injured cattle is,
where **0 represents the lowest impact** and **10 represents the highest financial impact**

	0	1	2	3	4	5	5	6	7	8	9
Number ()											

Q27 Using a scale of 0-10, the **financial impact** on you of **treating acutely injured cattle** is,
where **0 represents the lowest impact** and **10 represents the highest financial impact**

	0	1	2	3	4	5	6	7	8	9	10
Number ()											

Q28 Using a scale of 0-10, the **financial impact** on you of using **casualty slaughter** to manage acutely injured cattle is,
where **0 represents the lowest impact** and **10 represents the highest financial impact**

	0	1	2	3	4	5	6	7	8	9	10
Number ()											

Q29 My **decision making** in relation to the management of acutely injured cattle is influenced by (tick all that apply)

- ☐ My Vet
- ☐ Farmers
- ☐ My family
- ☐ Abattoir owners
- ☐ Farming Organisations
- ☐ Other (specify) ____

Q30 Does **OFES** have any **unrealised potential**?

- ○ Yes
- ○ No

Skip To: Q32 If Does OFES have any unrealised potential? = No

---

Q31 **If yes** can you outline **three ways this potential may be realised** so as to encourage the wider adoption of the procedure

- ☐ 1 ____
- ☐ 2 ____
- ☐ 3 ____

Q32 Is there **anything else** you would like to add in relation to the management of acutely injured cattle?

____

____

____

____

____

End of Block: Consent
